# RECOGNIZING AND ENGAGING FAMILY CAREGIVERS ACROSS THE CARE CONTINUUM: FULFILLING NATIONAL STRATEGY ACTIONS

**DOI:** 10.1093/geroni/igad104.1880

**Published:** 2023-12-21

**Authors:** Beth Fields, Lauren Bangerter, Lauren Bangerter

## Abstract

Millions of family caregivers (relatives, partners, or other support persons) provide support to older adults across the care continuum, but feel unrecognized and disengaged in health care and social service systems. To address this gap, congress passed the Recognize, Assist, Include, Support, and Engage (RAISE) Family Caregiving Act in January 2018. The RAISE Act established a Council to deliver recommendations to the Secretary of Health and Human Services on how to increase recognition and engagement of family caregivers. This Council released the National Strategy to Support Family Caregivers in September 2022, detailing recommended actions for health care and social service systems. In response to the recommended actions, this symposium will present innovative approaches to increase recognition and engagement of family caregivers across the care continuum. This symposium will help bridge the policy and practice gap by offering health care and social service systems examples of how to involve family caregivers across the care continuum. Dr. Riffin will describe a checklist-based intervention to support family caregivers in primary care. Drs Perpezko and Toto will discuss the convergence of goal setting among older adults and their family caregivers in the home setting. Ms. Muntefering will report facilitators and barriers to involving family caregivers in hospital care. Dr. Wyman will identify effective clinician behaviors for including family caregivers in geriatric mental health VA services. Dr. Werner will share an online portal for supporting family caregivers in rural areas.

